# The Optical and Electrical Performance of CuO Synthesized by Anodic Oxidation Based on Copper Foam

**DOI:** 10.3390/ma13235411

**Published:** 2020-11-27

**Authors:** Boyou Wang, Binhua Cao, Chen Wang, Yubo Zhang, Huifang Yao, Yongqian Wang

**Affiliations:** 1 Engineering Research Center of Nano-Geomaterials of Ministry of Education, Faculty of Materials Science and Chemistry, China University of Geosciences, 388# Lumo Road, Wuhan 430074, China; wby@cug.edu.cn (B.W.); wangchenii@163.com (C.W.); z7899zyb@126.com (Y.Z.); 2 Yantai Coastal Geological Survey Center, China Geological Survey, 287# Jichang Road, Yantai 264004, China; yxyang@cug.edu.cn; 3 Hubei Key Laboratory of Forensic Science, Hubei University of Police, 99# Nanniwan Avenue, Wuhan 430034, China

**Keywords:** Cu foam, anodic oxidation, CuO nanostructure, optical and electrical properties

## Abstract

Metal oxide semiconductor materials have a wide range of applications in the field of solar energy conversion. In this paper, CuO was prepared directly on copper foam substrate by anodic oxidation. The effects of current density and anodizing temperature on sample preparation and performance were studied. Field emission scanning electron microscopy (FESEM) and X-ray diffractometer (XRD) had been used to determine the morphology and phase structure of the sample, and its optical and electrical properties were discussed through UV-vis spectrophotometer and electrochemical tests. In addition, the influences of experimental conditions such as current density and reaction temperature on the morphology and properties of CuO were systematically discussed. The FESEM images showed that as the anodic oxidation temperature increase, the morphology of the prepared sample changed from nanowires to leaf-like CuO nanosheets. According to the results of XRD, the structure of prepared CuO was monoclinic, and the intensity of diffraction peaks gradually increased as anodizing temperature increased. We found that the optimum current density and anodizing temperature were 20 mA cm^−2^ and 60 °C, respectively. The results of electrochemical indicated that the CuO electrode based on copper foam (CuO/Cu foam) prepared at the optimum exhibited the highest specific capacitance (0.1039 F cm^−2^) when the scan rate was 2 mV s^−1^.

## 1. Introduction

Solar energy is a precious source of clean energy that has the advantages of being abundant, virtually pollution-free, and available in a variety of ways. Photothermal conversion, photoelectric conversion, and photochemical conversion are common ways to make full and effective use of solar energy [1,2]. Among many metal oxides, copper oxide (CuO) has attracted extensive attention, as a typical P-type semiconductor material and narrow band gap (1.2 eV–1.9 eV). CuO has the advantage of high visible light absorption efficiency, low corrosion resistance, good chemical stability, non-toxic, and easy preparation [3,4,5,6]. CuO has been extensively applied to catalytic reactions [7], antibacterial [8], gas-sensors [9], lithium-ion electrode materials [10], super-capacitors [11], solar cells [12], and other fields. The preparation methods of CuO nanostructures are also varied and mature. The commonly used preparation methods include thermal oxidation [13], anodization [14], wet-chemical [15], etc.

Metal foams are new functional materials that have been applied more and more widely in recent decades [16]. They are a kind of three-dimensional (3D) material with a large number of pores and a metal or metal alloy as the framework [17]. Because of the larger specific area, it has great application value in the field of catalysis [18]. Cao [19] reported the synthesis of stable lithium metal anode by in situ growth and chemical etching methods. The results indicated that due to the large specific surface area of CuO nanowires substrate, the stable Li metal anode exhibited high performance in reducing local current density. Carrera-Crespo [20] reported the effect of thermal oxidation on the CuO nanoneedles and its evaluation as photocathodes. La [21] used the method of electrochemical anodization to prepare wire-like bundle of Cu(OH)_2_. By changing the experimental conditions such as current density and electrolyte concentration, they found that the surface of the copper foil is only covered with a Cu_2_O layer at low current density (<0.8 mA cm^−2^). As the current density increase (>1 mA cm^−2^), the growth of Cu(OH)_2_ became more advantageous than the formation of CuO. Li [22] reported that the synthesis of CuO with different morphologies at a constant current density. The morphology of CuO was controlled by using different electrolyte solutions and annealing atmosphere. They found that the CuO/Cu foam nanosheets had smaller charge transfer resistance.

This work evaluated the influence of current density and anodizing temperature on the micromorphology and performance of samples. At room temperature, we succeeded in the synthesis of CuO/Cu foam nanowire arrays. When the reaction temperature rose above 40 °C, the morphology of CuO changed to nanosheets. Their optical absorption and chemical capacitance behavior were investigated. The prepared CuO was uniformly and densely distributed on the Cu foam substrate. Since the synthesized CuO was directly contact with Cu substrate, it was beneficial to reduce the resistance between CuO and Cu foam, thus increasing the utilization rate of CuO. The three-dimensional (3D) open network structure of Cu foam increased the contact area with the electrolyte solution and promoted the diffusion of the electrolyte. Thanks to the special structure accelerated the oxidation–reduction in CuO during electrochemical test, which was beneficial to enhance the electrochemical performance of the CuO/Cu foam [23,24]. Shinde S.K. [25] reported the synthesis of nanoflower-like CuO/Cu(OH)_2_ by SILAR method. The results showed that the maximum specific capacitance was 459 F g^−1^ in 2 mol dm^−3^ KOH electrolyte when the scan rate was 5 mV s^−1^.

## 2. Experimental

### Synthesis and Characterization of CuO Nanostructure 

In this experiment, all the chemicals such as KOH, HCl and NaOH purchased from Sinopharm Chemical Reagents Company. The commercial Cu foam was procured from Minquan Chemical Glass Testing Instrument Co Ltd. (Wuhan, China). The water-bath kettle was procured from Yuhua Instrument Co Ltd. (Gongyi, China). DC regulated power supply was procured from Hanshengpuyuan Technologies Co Ltd. (Beijing, China). All solutions of the configuration used deionized water as the solvent (conductivity 18.2).

The CuO nanostructure arrays were synthesized by anodizing Cu foam followed by annealing. The preparation process is briefly described as follows: For the purpose of removing the lipid and oxide layer that may exist on the surface of Cu foam, the pre-prepared commercial Cu foam (1.5 × 1.5 cm^2^) was ultrasonically cleaned in acetone and HCl solution and deionized water for 10 min in turn. The subsequent anodic oxidation process was carried out under a three-electrode system. In this system, the Cu foam as the anode, a platinum foil was used as the cathode and the silver chloride denotes the reference electrode. The electrolyte solution was KOH (2 mol dm^−3^) aqueous solution. The time for anodizing the Cu foam was 20 min. So as to explore the effect of reaction temperature on the morphology of the product, we used a thermostat to heat the electrolytic cell in a water bath. In order to achieve precise temperature control, we used a thermostat water-bath kettle (YUHUA HH-S2) for heating preservation. Before anodizing, the electrolytic cell would be placed in water bath for 10 min to ensure the system constant temperature. DC regulated power supply (HSPY-36-03) was used to control different current during the experimental. Subsequently the prepared precursor sample was repeatedly cleaned with absolute ethyl alcohol and deionized water, respectively, then placed in alumina boats annealed at 200 °C for 3 h in a baking oven to eventually successfully prepared the CuO/Cu foam. The effect of experimental parameters such as temperature and current density on the samples were investigated systematically.

FESEM (Japan Hitachi SU-8010) was used to observe the morphology of CuO/Cu foam prepared with different conditions. The phase structure was analyzed by XRD (Bruker) with Cu Kα radiation (λ = 0.154178 nm). The specific parameters of scanning rate were 0.02° s^−1^, and the 2θ range was 30–80°. The UV-vis absorption spectrum of CuO/Cu foam was investigated using an UV-vis spectrophotometer (Japan Shimadzu UV-2600). The electrochemical characteristics of the CuO/Cu foam electrodes were tested by an electrochemical workstation (China Chenhua CHI-660E). The test of cyclic voltammetry (CV), electrochemical impedance spectroscopy (EIS), and galvanostatic charge–discharge (GCD) were used to investigate the electrical performance of the CuO/Cu foam electrodes. A three-electrode system was used for the electrochemical performance. The working electrode was the CuO/Cu foam electrode with an effective area of 2 cm^2^, platinum foil (Pt 1 × 1 cm^2^) as the counter electrode, and the last one was silver chloride reference electrode (Ag/AgCl). The above tests were carried out in electrolyte solution of NaOH (5 mol dm^−3^) at room temperature. CV and GCD potential windows were 0–0.4 V.

## 3. Result and Discussion

### 3.1. The Effect of Current Density on CuO Morphology

**Figure 1:**
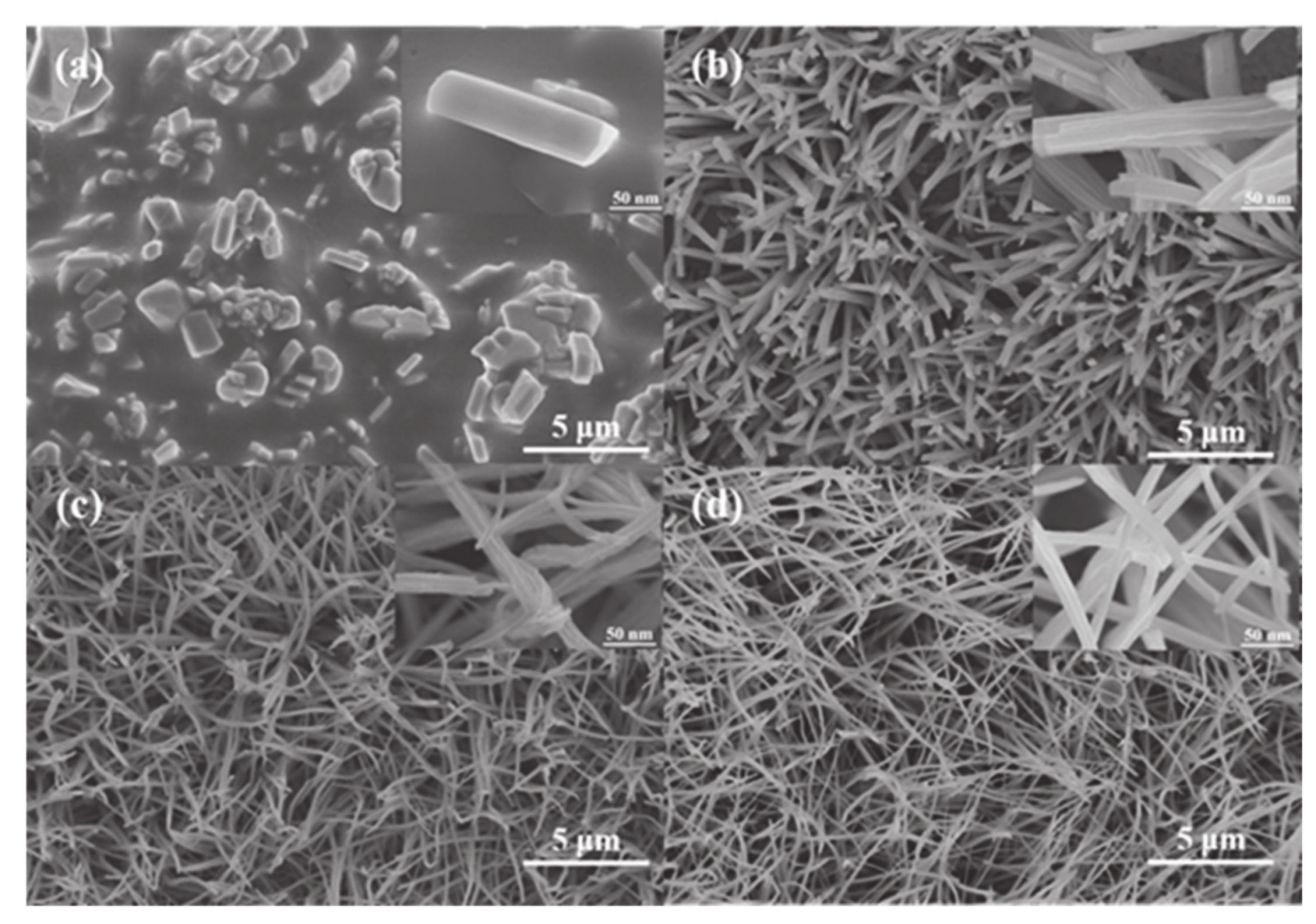
FESEM images of the samples prepared at room temperature with different current density, (**a**) 10, (**b**) 15, (**c**) 20, and (**d**) 25 mA cm^−2^.

For the purpose of studying the influence of different current densities on the morphology of samples, CuO nanostructures were obtained by applying anodized Cu foams with different current densities at room temperature and then by heat treatment. Figure 1 shows the FESEM images of CuO/Cu foam prepared at current density of 10, 15, 20, and 25 mA cm^−2^, respectively. Figure 1a clearly shows that the short rod-shaped CuO formed on the surface of Cu foam when the current density was 10 mA cm^−2^. As the current density increased, the length of CuO nanowires gradually increased. As can be seen from Figure 1d, the tips of the CuO nanowires gradually aggregated and tended to be shaped like a pagoda. However, according to the high magnification pictures, the diameter of a single nanowire is about 10–20 nm. It can be observed from Figure 1c that the CuO nanowire arrays were uniformly and densely dispersed on the Cu foam substrate.

### 3.2. The Effect of Anodizing Temperature on CuO Morphology

**Figure 2:**
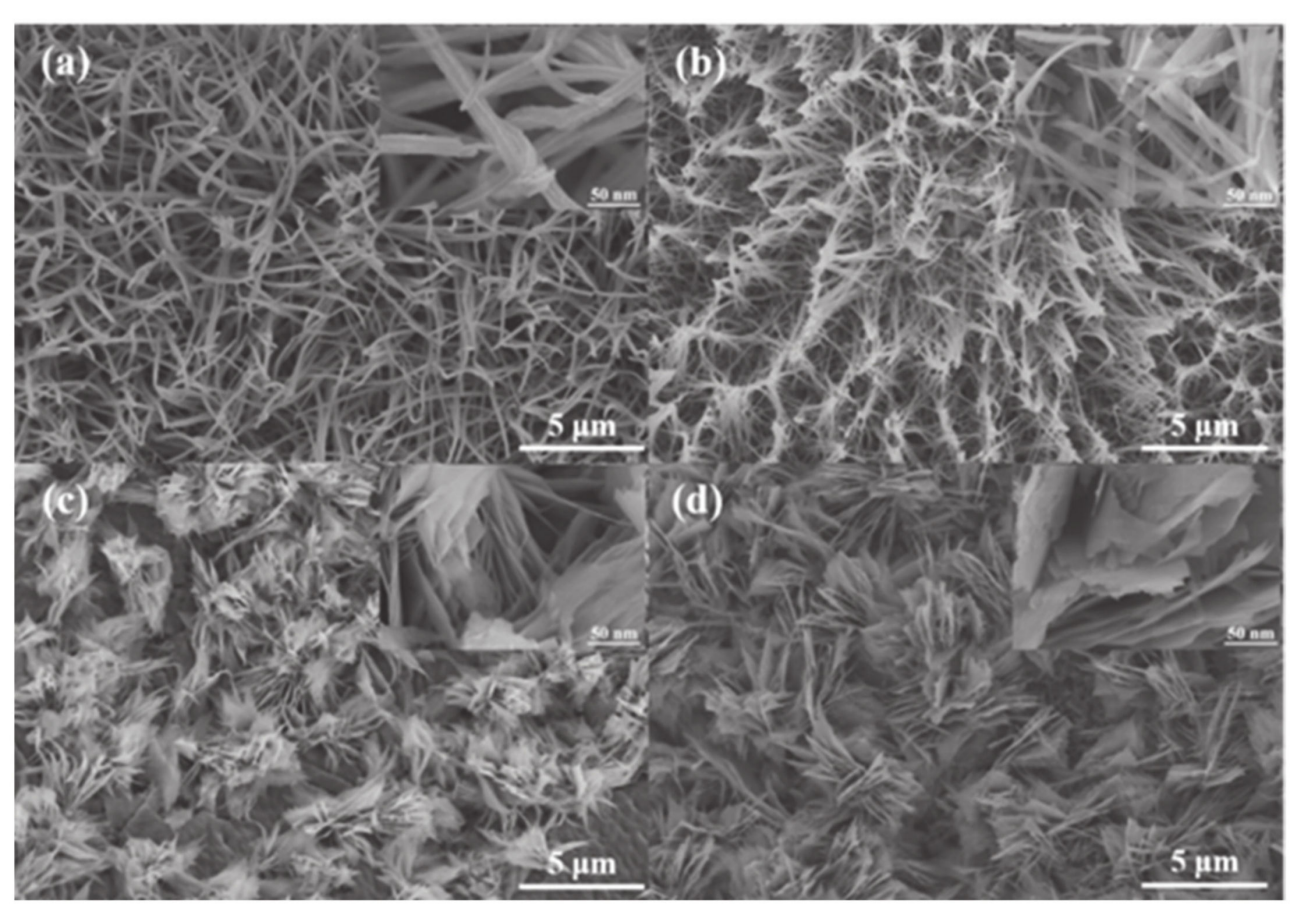
FESEM images of the samples prepared at 20 mA cm^−2^ current density with different anodizing temperature (**a**) 25, (**b**) 40, (**c**) 60, and (**d**) 80 ℃.

Figure 2 displays the high/low magnification FESEM images of CuO/Cu foam prepared at different anodizing temperature. It can be seen from the Figure 2 that when the anodizing temperature increased from room temperature to 40 ℃, CuO nanowires begin to gather at the top, being shaped like a pagoda. As the temperature continued to rise to 60 ℃, the morphology of CuO changed from nanowires to nanosheets. From Figure 2c,d, it can be observed that the Cu foam substrate was completely covered by the leaf-like CuO nanosheets. From the magnified images of FESEM, it can be clearly seen that a single nanosheets that composed of the leaf-like CuO structure was about 10 nm in thickness and 5 μm in length.

### 3.3. Phase Structure Analysis of CuO

**Figure 3:**
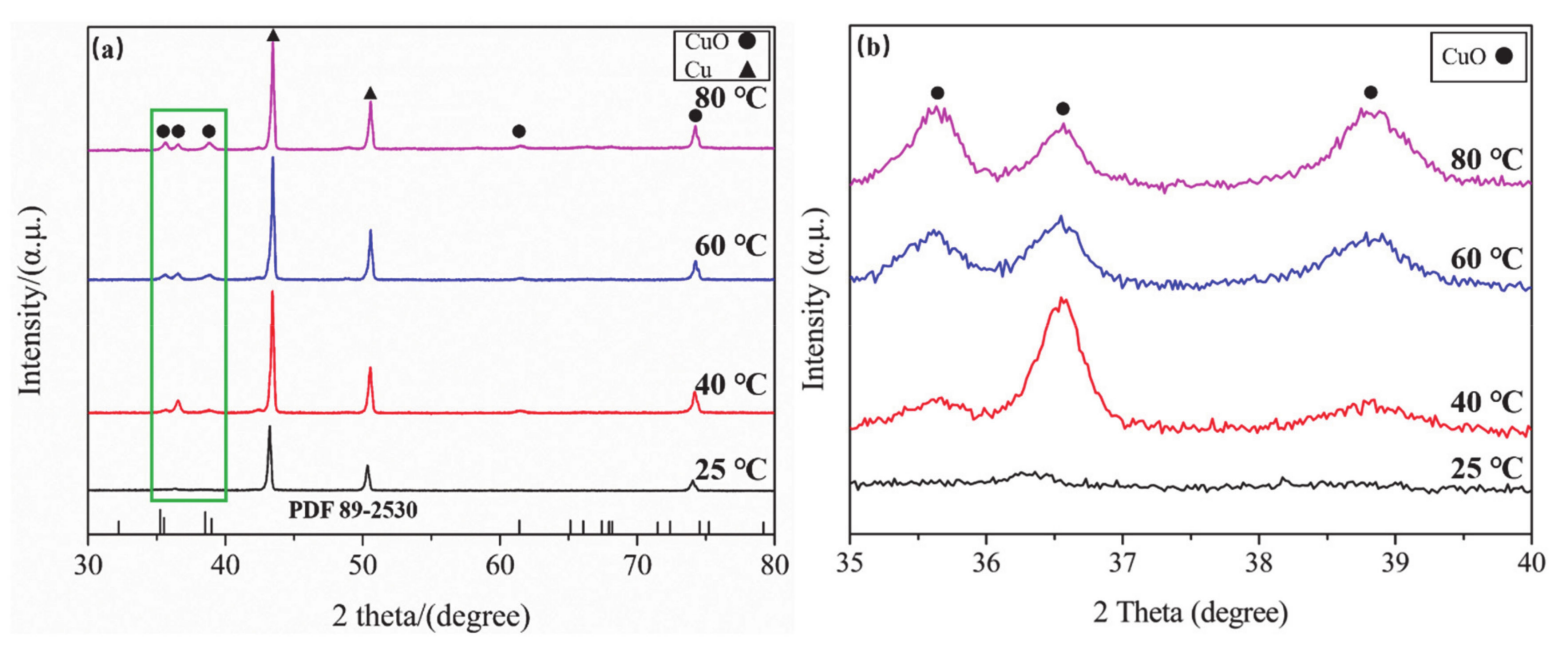
The XRD spectrum of CuO/Cu foam prepared with different anodizing temperature (**a**) and partial enlarged view (**b**).

Figure 3 shows the XRD patterns of the CuO/Cu foam prepared at 20 mA cm^−2^ current density with different temperatures. There are three strong reflection peaks at 2θ = 43.58, 50.51, and 74.21°, which can indicate the (1 1 1), (2 0 0), and (2 2 0) crystal planes of Cubic Cu structure (JCPDS No. 02-1225). The remaining reflection peaks could be identified as monoclinic CuO (JCPDS No. 89-2530) according to the search information. The specific parameters of the monoclinic CuO unit cell were a = 4.6839 Å, b = 3.4734 Å, c = 5.1226 Å. There are Cu peaks observed in the XRD spectrum. It is related to the thin structure of the synthesized CuO and the small size of the surface [26]. As can be seen from Figure 3, different diffraction peaks located at 2θ = 35.62, 38.64, and 39.09° corresponded to (0 0 2), (1 1 1), and (2 0 0) crystal planes respectively. Figure 3b, the partial enlarged view, clearly demonstrates the intensity of diffraction peaks at 35.62 and 39.09° gradually increased following elevated the reaction temperature. This indicates that the growth of CuO crystal nucleus is more favorable along (0 0 2) and (2 0 0) crystal planes, which also explains transition of the morphology of CuO in Figure 2.

### 3.4. UV-Vis Absorption Spectra of CuO

**Figure 4:**
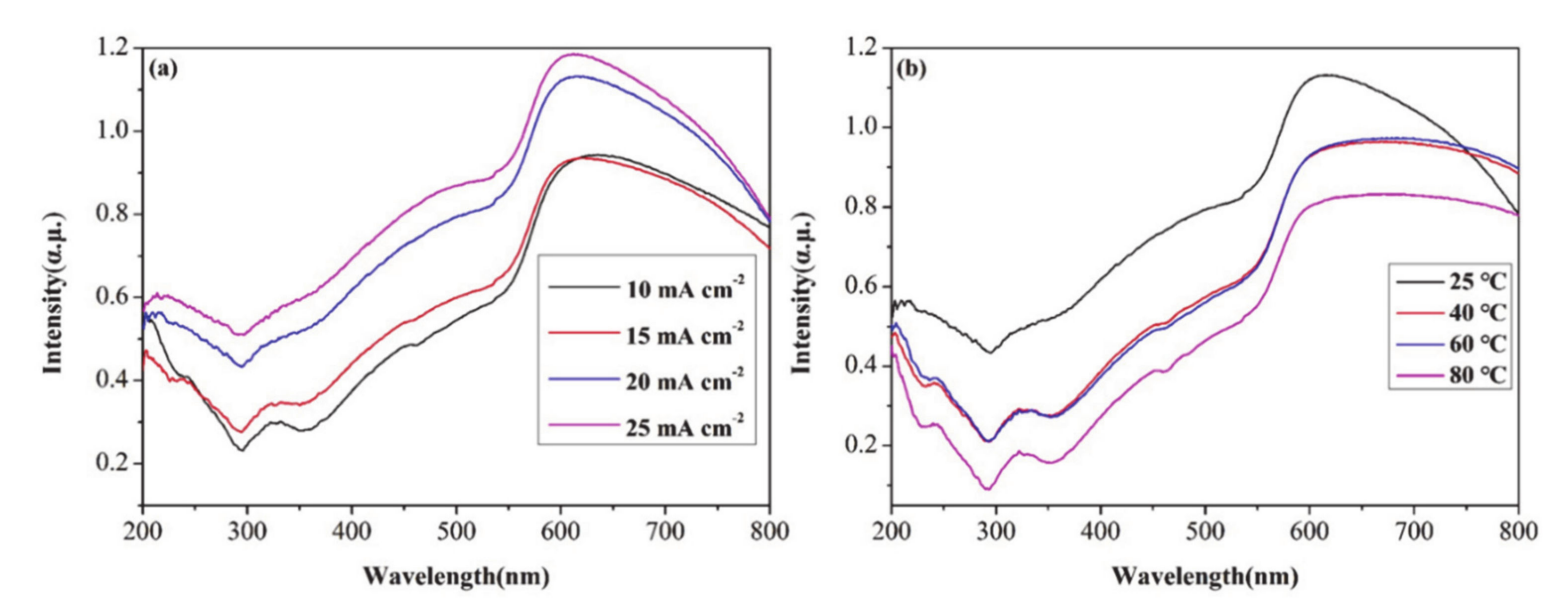
UV-vis absorption spectra of CuO/Cu foam synthesized with different conditions, different current density (**a**), and different anodizing temperature at 20 mA cm^−2^ (**b**).

Figure 4 shows the UV-vis spectra of CuO/Cu foam prepared at different current densities and anodizing temperatures. Figure 4 demonstrates that we could observe a good optical absorption spectrum in the wavelength of 200–800 nm, exhibiting good light absorption in the near ultraviolet and visible light regions. Figure 4a illustrates that the light absorption capacity of the samples enhanced as current density increases. However, as the anodizing temperature increases, the absorption spectrum decreases. This may be caused by the different methods of nucleation as the reaction temperature changes.

### 3.5. Properties of Electrochemical

**Figure 5:**
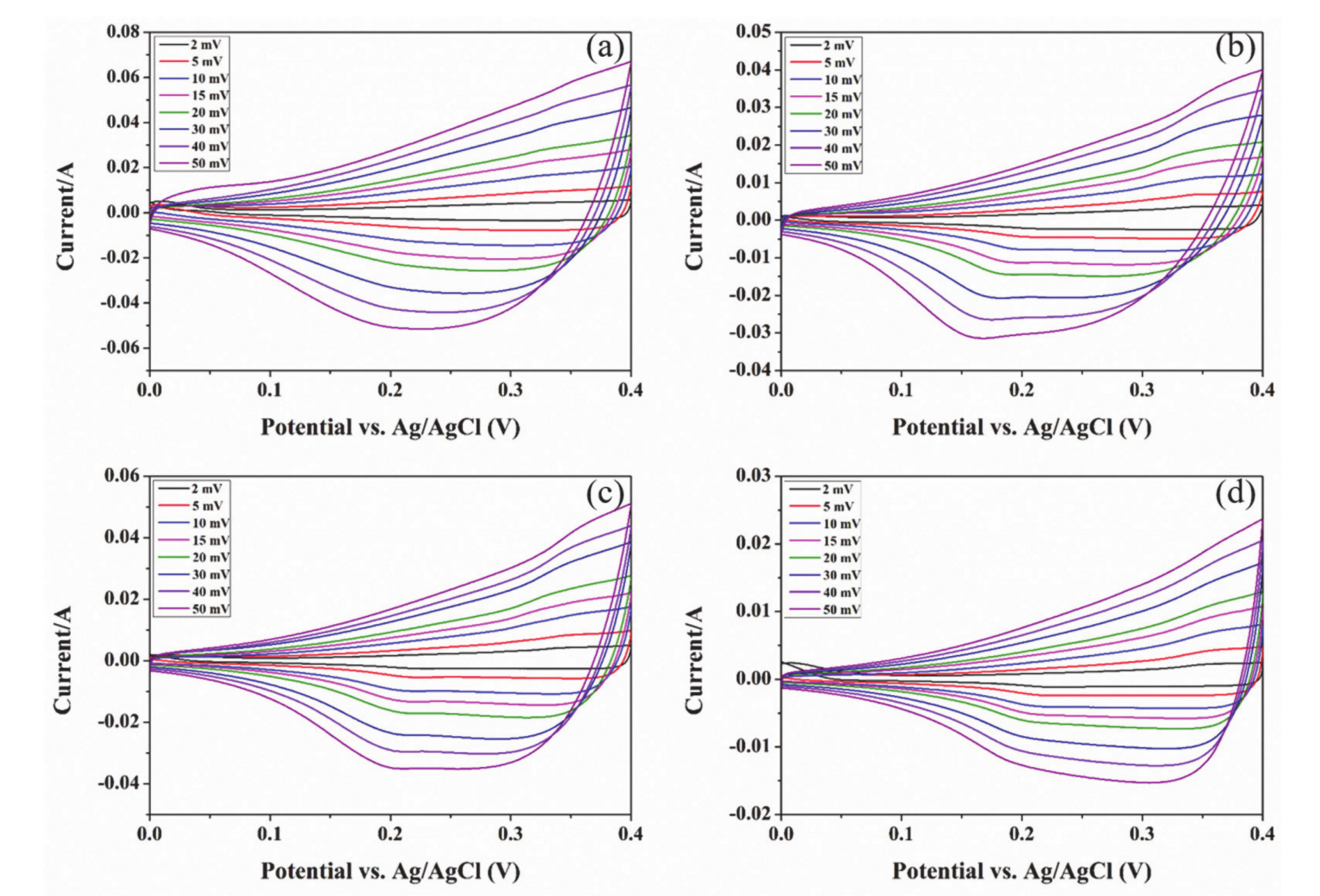
Cyclic voltammetry (CV) at different scan rates of the CuO/Cu foam prepared via different anodizing temperature (**a**) 25, (**b**) 40, (**c**) 60, and (**d**) 80 °C.

In this section, we will discuss in detail the electrochemical performance of the CuO/Cu foam electrode such as CV, EIS, and GCD. With the purpose of obtaining the best characteristic of the electrode, we immersed the CuO/Cu foam electrode in the electrolyte solution for 20 min at the beginning of measurement. Then, we performed a CV pre-scanning test on the CuO/Cu foam electrode at a voltage scanning rate of 20 mV s^−1^ lasting for 20 cycles. Figure 5 demonstrates the CV curves of the CuO/Cu foam prepared at 20 mA cm^−2^ current density with different anodizing temperatures, the scan rates are 2, 5, 10, 15, 20, 30, 40, 50 mV s^−1^, respectively. When testing the CV curves, the potential window is 0–0.4 V. The reason for choosing 0–0.4 V is that too high voltage will lead to electrolysis of water. It can be observed from the Figure 5 that each group of CV curves contains an insignificant oxidation peak and a broad reduction peak. It is completely different from the approximate rectangle CV curve of traditional Electric double layer capacitors (EDLCs), meaning that the electrochemical characteristic of the CuO/Cu foam electrode is based on the quasi-reversible and continuous Faraday redox reaction. Therefore, the pseudocapacitance characteristic of the CuO/Cu foam in alkaline solution were mainly determined by the redox reaction of Cu ions, which can be explained by Equations (1)–(4) [23]:

(1) $$\frac{1}{2}Cu_{2}O+OH^{-}↔CuO+\frac{1}{2}H_{2}O+e^{-}\;\;\;\;$$

(2) $$\frac{1}{2}Cu_{2}O+\frac{1}{2}H_{2}O+OH^{-}↔Cu{\left(OH\right)}_{2}+e^{-}\;$$

(3) $$CuOH+OH^{-}↔CuO+H_{2}O+e^{-}$$

(4) $$CuOH+OH^{-}↔Cu{\left(OH\right)}_{2}+e^{-}$$

The specific capacitance of the CuO/Cu foam electrodes were calculated according to Equation (5).

(5) $$C_{a}=\frac{\int I\left(V\right)dV}{2\upsilon S\Delta V}$$

*I* (V) represents the response current, *V* (V) is the relative potential of the Ag/AgCl electrode, *ν* denotes the sweep speed, *S* (cm^2^) is the effective area of contact between electrode and electrolyte solution during testing, and ∆*V* (V) is the value of the potential during the test. The specific capacitance of CuO/Cu foam electrode as shown in Table 1. 

**Figure 6:**
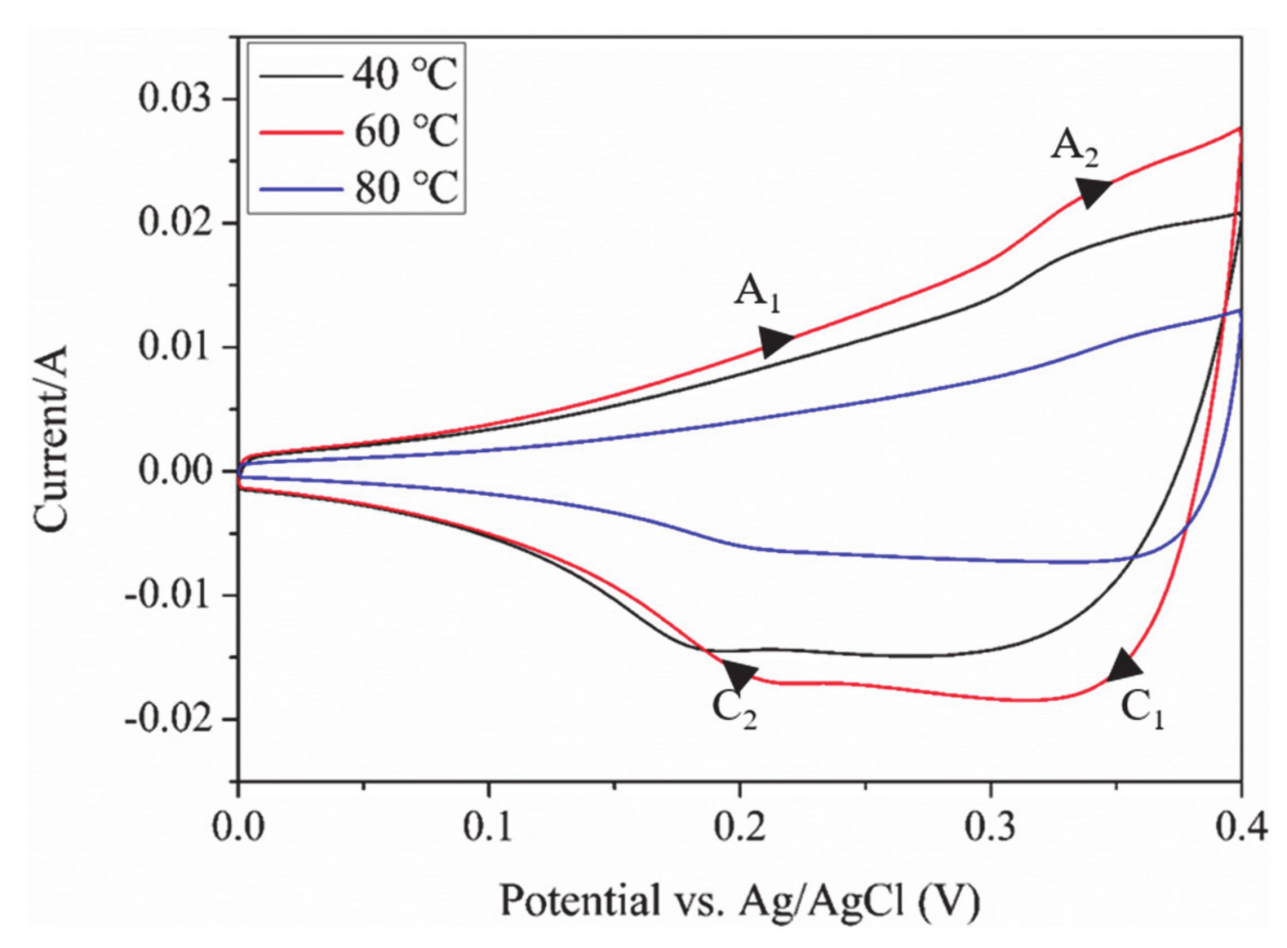
CV of the electrodes prepared with different anodizing temperature at 20 mV s^−1^ scan rate.

Figure 6 shows the CV curves of electrodes synthesized with different anodizing temperatures. The scan rate of CV test is 20 mV s^−1^. According to the calculation of Equation (5), Ca of the CuO/Cu foam electrodes are 0.048, 0.059, and 0.025 F cm^−2^, respectively. It can be seen from Figure 6 that Ca increased with the increase in anodizing temperature, until reaching its maximum when anodizing temperature reached 60 ℃, but it started to decrease with the increase in temperature. These phenomena can be attributed to the influence of the electrochemically active substance on CuO/Cu foam electrode. At first, as the anodizing temperature increased, the active material on the electrode increased and the leaf-like structure increased the contact area and promoted the improvement of electrochemical performance. However, when the total amount of active substance is too high, the contact between the electrolyte and electrode surface will be inhibited, resulting in a decrease in specific capacitance [24]. The anodic peak A1 and A2 arises from the process by which Cu_2_O (or Cu(OH)) is oxidized to CuO. The reduction process of CuO or Cu(OH)_2_ may be the reason why occurs the cathodic peak C1 and C2 [23]. All redox reaction process during the CV test can be explained by Equations (1)–(4).

**Figure 7:**
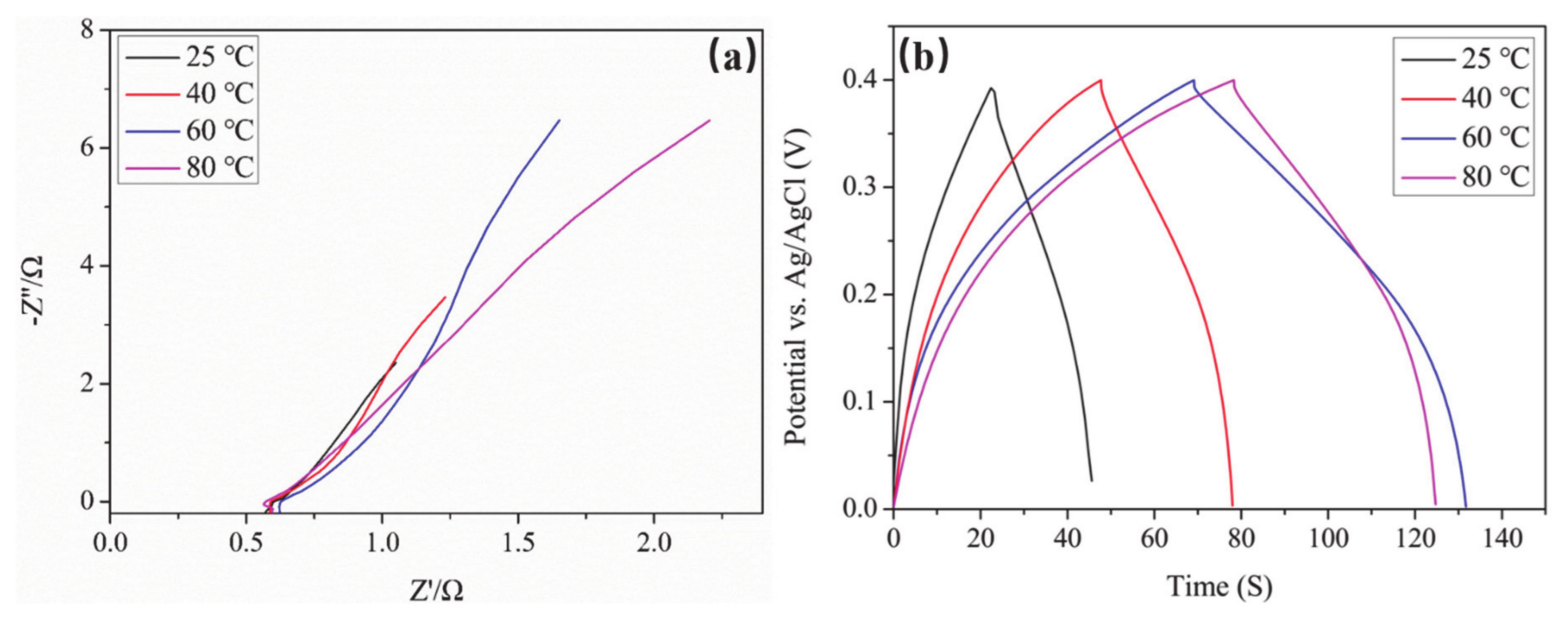
EIS of the CuO/Cu foam prepared with different anodizing temperature at 20 mA cm^−2^ current density (**a**) and GCD curves (**b**).

Figure 7a shows the EIS diagram of CuO/Cu foam electrodes in 5 M NaOH electrolyte solution. In an ideal state, the curve of impedance is nearly semicircular in the range of high frequency. It is mainly caused by the oxidation–reduction reaction in Equations (1)–(4). The semicircular arc in the range of high frequency shrinks, indicating that the transfer process of electron charge is rapid. The larger the radius, the greater the transfer resistance [14]. It can be seen from Figure 7a that as the anodizing temperature increases, the charge transfer resistance first decreases, but when the temperature continues to increase from 60 °C, the resistance increases instead. The transfer resistance of the CuO/Cu foam electrodes in the electrolyte is consistent with the results of specific capacitance in Table 1. However, it can be seen that the slope of the curve is relatively small in the low frequency region. The ideal capacitor should be a vertical line. The smaller slope was caused by the existence of Warburg impedance when the electrolyte solution diffuses inside the electrodes [11]. Figure 7b shows the GCD diagram of electrodes synthesized by different anodizing temperature at 20 mA s^−1^ current density. In order to understand the rate performance of the electrodes, the galvanostatic charge-discharge (GCD) curve was discussed. The GCD curves are evaluated to understand the rate capability of CuO/Cu foam electrode. The shape of GCD curves in the Figure 7b is obviously different from the triangular GCD curves of EDLCs. The smooth and symmetrical GCD curves of CuO/Cu foam electrodes during the process of charging and discharging show the excellent pseudocapacitance characteristics. As shown in Figure 7b, with the anodizing temperature increase, the time of charge–discharge increases first and then decreases. The GCD curves are not straight line during the process of charging and discharging, which means that the reaction is Faraday redox reaction. The conclusion of GCD is the same as CV.

### 3.6. Growth Mechanism of CuO/Cu Foam

**Figure 8:**
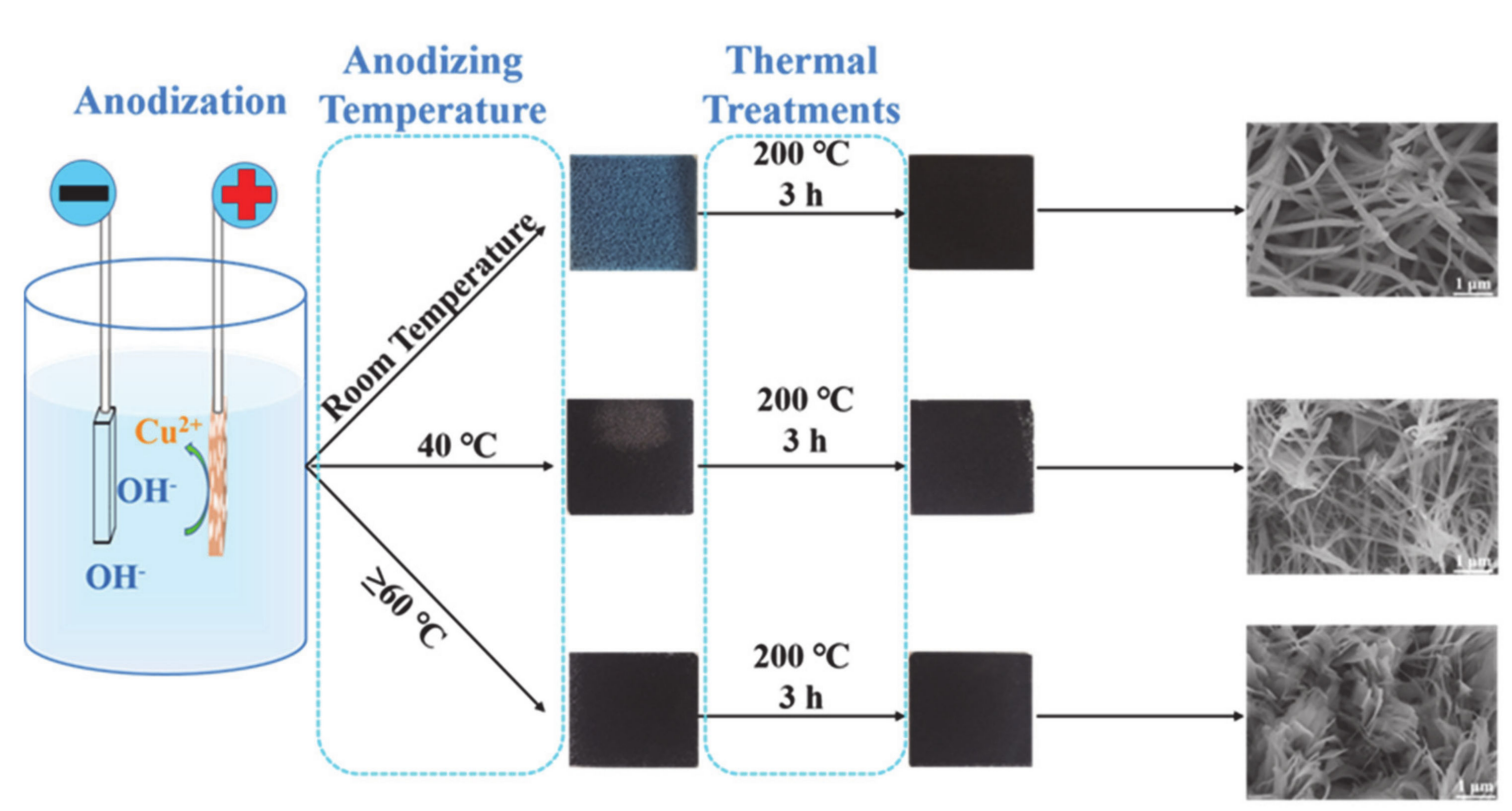
The synthesis process of CuO/Cu foam electrode.

**Scheme 1:**
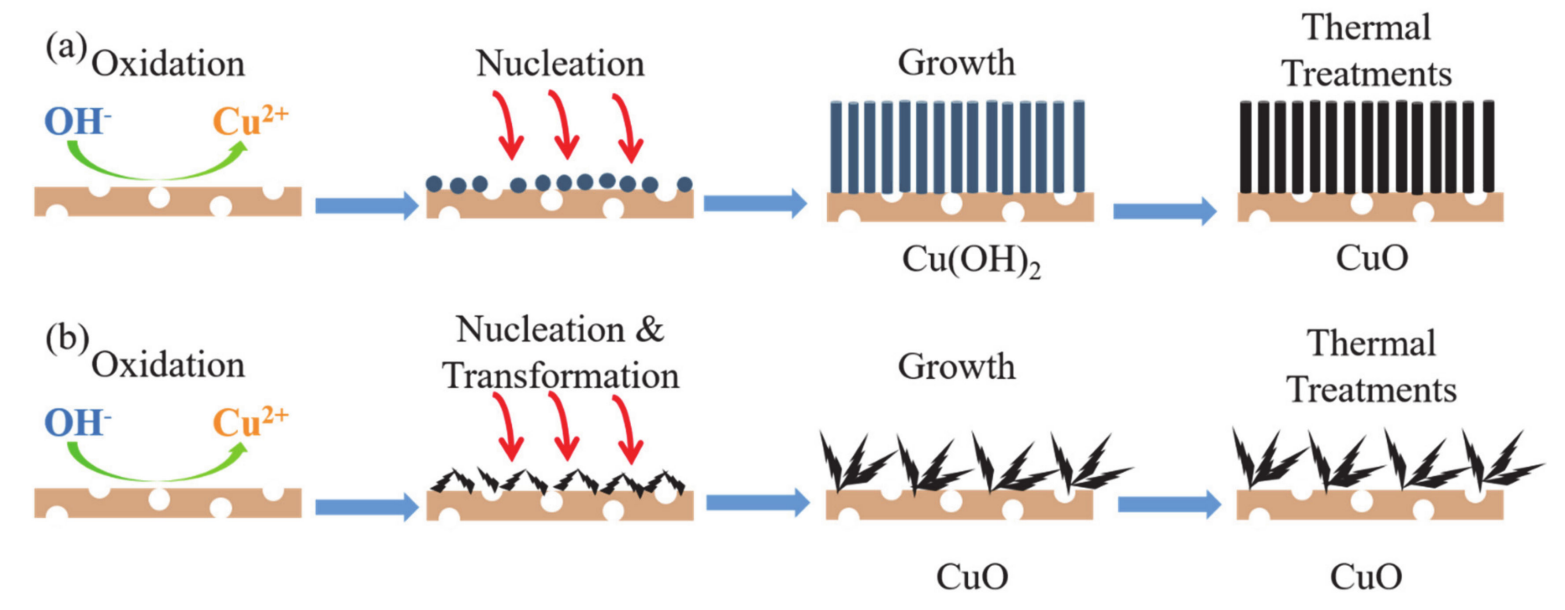
The growth mechanism of CuO nanostructures. (**a**) at room temperature, (**b**) above 40 °C.

The synthesis principle of the CuO/Cu foam electrodes prepared by anodic oxidation was shown in Figure 8 and Scheme 1. The anodic oxidation process can be briefly described as: immerse the Cu foam in 2 mol dm^−3^ KOH electrolyte and apply a constant current for 20 min. At room temperature, the Cu foam substrate was oxidized, and the ions of Cu^2+^ and Cu^+^ occurred when applying a constant current. The ions were released into the KOH electrolyte. At the same time, Cu^2+^ was captured by the OH^-^ in the solution to generate a very small Cu(OH)_2_ nucleus. Meanwhile, the Cu(OH)_2_ nucleus can grew on and on along the (1 1 1) crystal plane in strong alkaline conditions as the anodizing time and current density increased, finally formed the structure of Cu(OH)_2_ nanowire arrays [27]. After calcining at 20 °C for 3 h, Cu(OH)_2_ was dehydrated and converted into CuO nanowire arrays, as shown in Scheme 1a. This is also the reason why nanowires gradually aggregate at the top as the current density increase in Figure 1. Scheme 1b demonstrates the growth process of leaf-like CuO nanosheets. At first the formation process of Cu(OH)_2_ nucleus was the same as above. However, when the reaction temperature rose (above 40 °C), the Cu(OH)_2_ nucleus was more likely dehydrated and formed the CuO nucleus. This is the reason why Cu foam become black rather than blue after anodization, when the reaction temperature rises above 40 °C. Then, the CuO nucleus continuously grew along the (0 0 2) and (2 0 0) crystal planes until it formed leaf-like CuO nanosheets, which explained the transformation of CuO morphology in Figure 2 [28,29]. In order to ensure a complete reaction, the precursor was heat-treated under the condition of heating at 200 ℃ for 3 h in an air atmosphere and ultimately forms CuO nanostructures on the Cu foam substrate [30,31,32,33,34]. As shown in the figure below, the color of the Cu foam surface became black at the end of experiment. The entire reaction can be explained by the following Equations (6)–(11):

(6) $$Cu\overset{anodize}{\to }Cu^{2+}+2e^{-}$$

(7) $$Cu\overset{anodize}{\to }Cu^{+}+e^{-}$$

(8) $$Cu^{2+}+2OH^{-}\overset{}{\to }Cu{\left(OH\right)}_{2}$$

(9) $$Cu^{+}+OH^{-}\overset{}{\to }CuOH$$

(10) $$Cu{\left(OH\right)}_{2}\overset{\Delta }{\to }CuO+H_{2}O$$

(11) $$CuOH\overset{\Delta }{\to }\frac{1}{2}Cu_{2}O+\frac{1}{2}H_{2}O$$

## 4. Conclusions

It was illustrated that the CuO/Cu foam nanostructures could be successfully synthesized by anodic oxidation. It was proved that the optimum current density and anodizing temperature were 20 mA cm^−2^ and 60 °C, respectively. When the reaction temperature rose (above 40 °C), the Cu(OH)_2_ nucleus was more likely dehydrated and formed the CuO nucleus. The results of XRD showed the CuO nucleus continuously grew along the (0 0 2) and (2 0 0) crystal planes, which explained the transformation of CuO morphology. The growth mechanism of CuO/Cu foam had been shown in Scheme 1. We also found that the different morphologies of the prepared CuO nanostructures had an impact on its photoelectric properties. The photoelectric performance of CuO/Cu foam also was investigated. It can be identified that the light absorption of CuO was not good near the wavelength of UV according to the Figure 4 of UV-vis spectrum. Therefore, subsequent composite modification should be considered to improve its absorption of UV-vis light. The shape of CV and GCD curves revealed the electrochemical characteristic of the CuO/Cu foam electrode was based on the quasi-reversible and continuous Faraday redox reaction. The CuO/Cu foam electrode prepared at the optimum exhibited the highest specific capacitance (0.1039 F cm^−2^) when the scan rate was 2 mV s^−1^. In view of the advantages of low-cost, high specific surface, and excellent optical and electrical performance, CuO/Cu foam may have broad application prospects in the fields of catalysts, sensors, and so on.

## Figures and Tables

**Table 1:** Specific capacitance of CuO/Cu foam electrodes.

Scan Rates (mV s^‒1^)	Temperature (°C)		
	40	60	80
2	0.093263	0.103937	0.049606
5	0.069931	0.082675	0.037224
10	0.058045	0.072527	0.030095
15	0.052582	0.063057	0.027631
20	0.048352	0.059108	0.025253
30	0.04282	0.053433	0.022761
40	0.039119	0.04663	0.020696
50	0.03561	0.042637	0.019347
